# Degradative Ability of Mushrooms Cultivated on Corn Silage Digestate

**DOI:** 10.3390/molecules25133020

**Published:** 2020-07-01

**Authors:** Stefano Fornito, Federico Puliga, Pamela Leonardi, Michele Di Foggia, Alessandra Zambonelli, Ornella Francioso

**Affiliations:** 1 Department of Agricultural and Food Sciences, University of Bologna, Viale G. Fanin 40, 40127 Bologna, Italy; stefano.fornito93@gmail.com (S.F.); pamela.leonardi@unibo.it (P.L.); alessandr.zambonelli@unibo.it (A.Z.); ornella.francioso@unibo.it (O.F.); 2 Department of Biomedical and Neuromotor Sciences, University of Bologna, Via Belmeloro 8/2, 40126 Bologna, Italy; michele.difoggia2@unibo.it

**Keywords:** corn digestate, white-rot fungi, lignin degradation, ATR-FTIR, mushroom cultivation

## Abstract

The current management practice of digestate from biogas plants involves its use for land application as a fertilizer. Nevertheless, the inadequate handling of digestate may cause environmental risks due to losses of ammonia, methane and nitrous oxide. Therefore, the key goals of digestate management are to maximize its value by developing new digestate products, reducing its dependency on soil application and the consequent air pollution. The high nitrogen and lignin content in solid digestate make it a suitable substrate for edible and medicinal mushroom cultivation. To this aim, the mycelial growth rate and degradation capacity of the lignocellulosic component from corn silage digestate, undigested wheat straw and their mixture were investigated on *Cyclocybe aegerita, Coprinus comatus, Morchella importuna, Pleurotus cornucopiae* and *Pleurotus ostreatus*. The structural modification of the substrates was performed by using attenuated total reflectance-Fourier transform infrared (ATR-FTIR) spectroscopy. Preliminary in vitro results demonstrated the ability of *P. ostreatus*, *P. cornucopiae* and *M. importuna* to grow and decay hemicellulose and lignin of digestate. Cultivation trials were carried out on *C. aegerita*, *P. cornucopiae* and *P. ostreatus*. *Pleurotus ostreatus* showed the highest biological efficiency and fruiting body production in the presence of the digestate; moreover, *P. ostreatus* and *P. cornucopiae* were able to degrade the lignin. These results provide attractive perspectives both for more sustainable digestate management and for the improvement of mushroom cultivation efficiency.

## 1. Introduction

Managing organic waste streams is a major challenge for the agricultural industry. Anaerobic digestion (AD) treatment process is considered the most suitable bioenergy technology to treat wastes for biogas production from agriculture, industry and household food wastes [1]. Public policies of several EU Member States have promoted the use of AD to treat organic wastes and to generate renewable energy. According to the estimates, about one-third of the EU’s 2020 target for renewable energy in transport could be met by using biogas produced from biowaste, while around 2% of the EU’s overall renewable energy target could be met if all biowaste was turned into energy [2].

Digestate is a heterogeneous material produced in large amounts during the AD process [3]. The physicochemical characteristics of digestate depend on the nature and composition of feedstocks, as well as on the operational parameters of the process. Agricultural wastes typically have a high content of lignocellulose. In this rigid structure, lignin coats cellulose and hemicellulose while blocking their degradation by anaerobic bacteria [4,5]. Pretreatment methodologies utilizing energy-intensive processes (high pressures and temperatures) and harsh chemical compounds (NaOH and H_2_SO_4_) are currently used, in order to better perform their utilization in the AD process [6,7].

One of the main questions about the reuse of digestate is how to prevent nutrient imbalances in the receiving environment. In agriculture, digestate is considered a useful nutrient source, but its use is influenced by the disposal limits for N and P [8]. Moreover, the digestate used as a fertilizer is still a source of greenhouse gases (GHG). These gases can be produced and emitted during digestate storage and during its spreading upon the field, although their impact is relatively lower than an untreated biomass [9]. However, the ammonia release and nitrate leaching are still a critical point with respect to N_2_O and CH_4_ emissions from digestate [10].

Therefore, new strategies and alternative uses of digestate are necessary in view of the growth of biogas plants [11].

Lignocellulolytic mushrooms are an attractive resource that allows the biotransformation of lignocellulosic wastes into a value-added bioproduct [12]. White-rot mushrooms such as *Cyclocybe aegerita* (V. Brig.) Vizzini, *Ganoderma lucidum* (Curtis) P. Karst., *Ganoderma resinaceum* Boud., *Lentinula edodes* (Berk.) Pegler, *Pleurotus cornucopiae* (Paulet) Rolland, *Pleurotus ostreatus* (Jacq.) P. Kumm. and *Schizophyllum commune* Fr. are the most effective mushrooms for delignification due to the production of a large variety of ligninolytic extracellular enzymes such as laccase, lignin manganese and versatile peroxidases, galactose oxidase, glyoxal and alcohol oxidase, benzoquinone reductases and lytic polysaccharide monooxygenases [13,14,15]. On the other hand, brown root mushrooms such as *Laetiporus sulphureus* (Bull.) Murrill, some coprophilous mushrooms like *Coprinus comatus* (O.F. Müll.) Pers. and the post-fire mushroom *Morchella importuna* M. Kuo, O’Donnell & T.J. Volk have been shown to also be able, although to a lesser extent, to produce oxidative enzymes for lignin degradation [13,15,16,17]. Most of these mushrooms are edible and/or have medicinal proprieties and have been successfully cultivated [18,19,20]. *Coprinus comatus* is also cultivated on a biogas residue mixture and is patent-protected [21]. Only *M. importuna* has begun to be cultivated recently, predominately in open fields in China [22].

In the past, several analytical approaches were carried out to study the ability of fungal species to decompose lignin. Since lignin is closely associated to cellulose and hemicellulose, its separation is considered problematic, because the extraction procedures take time and lead to chemical and structural changes caused by condensation and oxidation reactions [23]. There are two main conventional techniques to separate lignin from hemicellulose and cellulose: the first is to remove cellulose and hemicellulose, leaving most of the lignin as a solid residue, and the second is to extract lignin using fractionation procedures, leaving the other components [24]. As a result, the original structural characteristics of lignin may be missing.

Attenuated total reflectance-Fourier transform infrared (ATR-FTIR) spectroscopy is a nondestructive technique that has been successfully used for detecting chemical compounds present in complex mixtures, because each molecule is characterized by a specific spectrum. Pandey and Pitman [25] and Gupta et al. [26] have widely used this technique to monitor the chemical variations that occur during wood degradation by chemical and biological treatments. However, some spectra may appear poorly resolved due to the existence of highly overlapping and hidden peaks. Consequently, it is not possible to assign specific peaks to the vibrations of specific functional groups. In such cases, the application of a curve-fitting analysis has become an important tool for qualitative and quantitative analyses of IR spectra [27].

In this context, the main goal of the project was to use mushroom cultivation for degrading the ligninolytic fraction of digestate. Several edible and medicinal mushroom species were tested, and their ability to degrade the lignin fraction was assessed by using ATR-FTIR spectroscopy.

## 2. Results

### 2.1. In Vitro Mycelial Growth and Elemental Analysis of C and N of Substrates

*Ganoderma lucidum, G. resinaceum, L. sulphureus* and *L. edodes* did not grow or grew very slowly on the digestate, whereas *C. aegerita*, *M. importuna*, *P. cornucopiae* and *P. ostreatus* grew similarly or significantly more (*P. ostreatus*) on the corn digestate (CD) compared to the straw (Table S1 and Figure S1). For this reason, the C and N content and ATR-FTIR spectra were carried out only on the latter four species. *Coprinus comatus* was also included, because, recently, a substrate containing biogas residues has been patented for its cultivation [21].

Table 1 shows the C and N values determined in CD, corn digestate 50%-wheat straw 50% (CD-WS) and wheat straw (WS) before inoculation (control) and after 12 d of mycelial growth. The C percentage of CD-WS showed no intermediate value due to the high chemical complexity of CD. The C content of substrates inoculated with several mushrooms showed no considerable variation (Table 1). The C content was significantly lower in *C. aegerita* on CD and significantly higher in *C. comatus, M. importuna, P. cornucopiae* and *P. ostreatus* on WS than in the untreated substrates (control).

The nitrogen content on WS inoculated with the different mushrooms was generally significantly lower than the control (Table 1). These changes led to a significant increase in the C/N ratio on WS, which was 31 in the control and ranged from 59 to 69 after the fungal growth (Table S2). On the CD substrate, there were no significant differences between treatments, although a slight decrease of N was apparent in the presence of *C. comatus* and *P. cornucopiae* (Table 1). In the CD-WS substrate, only *C. aegerita, P. ostreatus* and *M. importuna* significantly decreased the N content. No variation was observed for the other species. Considering the C/N ratio, no variation was observed on the CD, while on the CD-WS, the C/N ratio was 34 in the control and 49 after the growth of *P. ostreatus* (Table S2).

### 2.2. ATR-FTIR of Substrates After In Vitro Mycelial Growth

**Figure 1:**
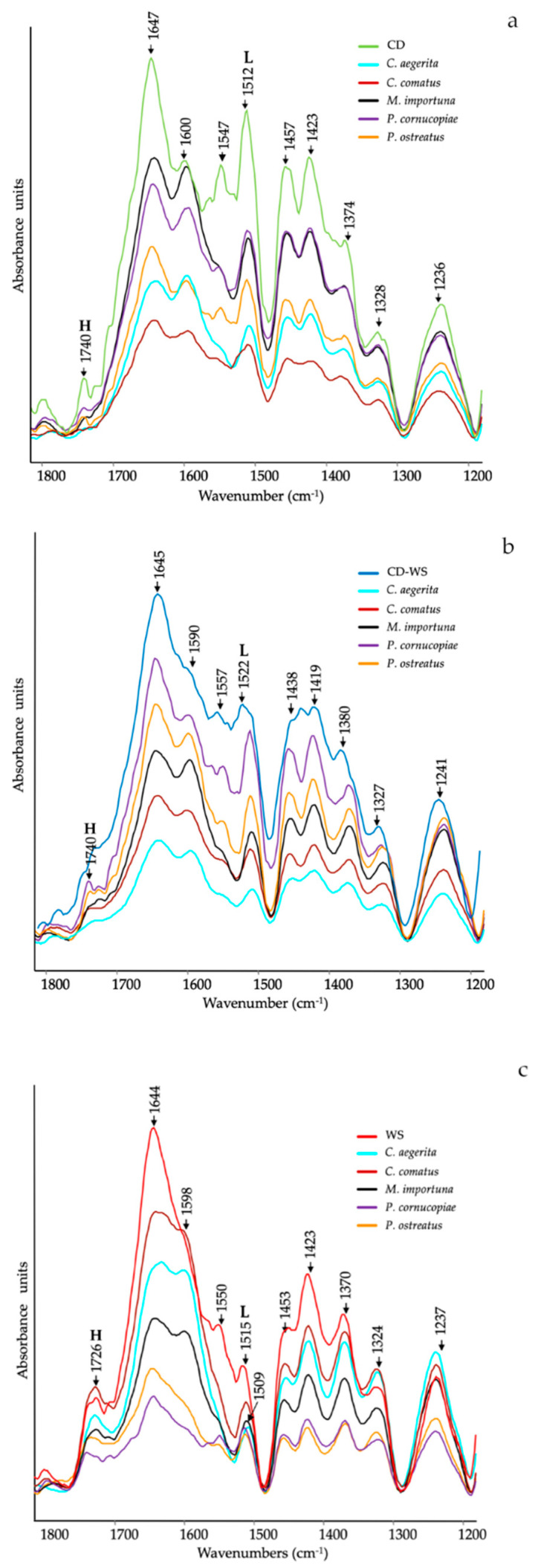
Attenuated total reflectance-Fourier transform infrared (ATR-FTIR) spectra of (**a**) corn digestate (CD, green line), (**b**) corn digestate 50%-wheat straw 50% (CD-WS, blue line) and (**c**) wheat straw (WS, red line) after 12 d of mycelial growth of *Cyclocybe aegerita* (cyan line), *Coprinus comatus* (dark red line), *Morchella importuna* (black line), *Pleurotus cornucopiae* (purple line) and *Pleurotus ostreatus* (orange line). **H** = hemicellulose and **L** = lignin (as references for lignin: [29,30,31]).

Figure 1 shows the ATR-FTIR spectra of the substrates (CD, CD-WS and WS) after 12 d of mycelial growth with different mushrooms species. The ATR-FTIR spectra clearly showed a significant structural variation of the main functional groups in the region between 1800–1200 cm^−1^, in which many clearly defined peaks provided information on their modification during degradation.

The major peaks can be listed as follows: 1740 cm^−1^ is ascribed to unconjugated C=O in xylans (hemicellulose) or in the heterocyclic cellulosic rings [28]; 1727 cm^−1^ is due to H-bonded acid/ketone carbonyl groups; ~1645 cm^−1^ is assigned to C=O stretching in conjugated ketones, water and amide I (proteins); ~1590 and 1510 cm^−1^ are attributed to aromatic skeletal vibrations (C═C) and aromatic breathing in lignin, respectively [29,30,31]; ~1547 cm^−1^ is related to amide II (proteins); 1457 cm^−1^ is due to C–H-bending in lignin and hemicelluloses and 1423 and 1370 cm^−1^ are assigned to CH_2_ and CH_3_-bending vibrations, respectively. These bands are typical of the mixture of both crystalline and amorphous cellulose [32]; 1318 cm^−1^ is attributed to C-H bending in crystallized cellulose I [33], and ~1240 cm^−1^ is assigned to the syringyl ring in lignin and C–O stretching in xylan of hemicellulose [25,34].

In more detail, the ATR-FTIR spectra of the CD substrate after mycelium growth, as compared to untreated CD (Figure 1a), showed a significant decrease in the relative intensity of the bands assigned to hemicellulose (1740 cm^−1^ and 1236 cm^−1^), proteins (1647 cm^−1^ and 1547 cm^−1^) and lignin (1600 cm^−1^ and 1512 cm^−1^). All these compounds changed in relation to the inoculated mushroom species.

In some instances, ester bands in hemicellulose have disappeared in CD treated with *C. aegerita* and *C. comatus*, as well as amide (II) in proteins, as supported by the slight reduction in total nitrogen (Table 1). Concerning the lignin bands (1600 cm^−1^ and 1512 cm^−1^), they progressively decreased in the series: *C. comatus < C. aegerita* < *P. ostreatus < P. cornucopiae < M. importuna*.

In the case of the ATR-FTIR spectra of the CD-WS substrate after mycelium growth, a slight difference compared to the untreated substrate was observed (Figure 1b). The relative intensities of hemicellulose bands considerably decreased in the substrate treated with *C. aegerita* and *C. comatus*, as well as in the protein bands (1645 cm^−1^ and 1557 cm^−1^)*,* although no correspondence with the total N was found. Regarding the peaks taken as a reference of lignin (1590 cm^−1^ and 1522 cm^−1^), they progressively decreased in the series: *C. aegerita < C. comatus < M. importuna < P. ostreatus <P. cornucopiae*.

Figure 1c showed the ATR-FTIR spectra of the WS substrate. As already observed on CD and CD-WS, the intensity of the hemicellulose bands (1726 cm^−1^ and 1237 cm^−1^) decreased in treatments with *P. cornucopiae* and *P. ostreatus*. As regards to the amide bands, a considerable reduction appeared in all treatments, as also supported by the variation of the total N (Table 1). The intensity of the band at 1509 cm^−1^ (lignin) was significantly reduced in all spectra, although it was more consistent in *P. ostreatus* and *P. cornucopiae*.

### 2.3. Fruiting Body Production

For the cultivation test, *C. aegerita*, *P. cornucopiae* and *P. ostreatus* were used. The choice was made based on the results obtained from the measurements of the mycelial growth rate on the CD (Table S1) and the commercial potential of the species. The species *M. importuna*, even though it has rapid development in in vitro tests, was not selected for cultivation trials, as its production of fruiting body is still difficult in controlled conditions [35].

**Figure 2:**
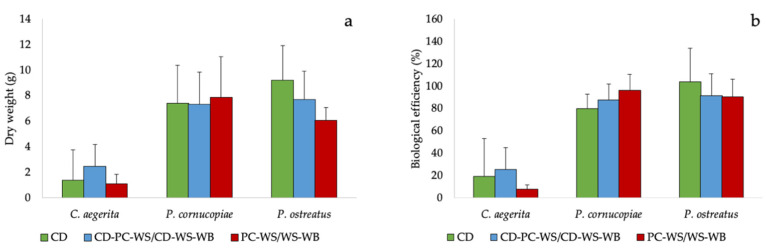
(**a**) Fruiting body production and (**b**) biological efficiency of the tested mushrooms species on the CD and on corn digestate-poplar chips-wheat straw (CD-PC-WS) or corn digestate-wheat straw-wheat bran (CD-WS-WB) and on PC-WS/WS-WB.

The different species used during the cultivation tests showed a fruiting body production (Figure 2a and Figure S2) and a biological efficiency similar in the different substrates used. Although there were no statistically significant differences between substrates, *P. ostreatus* grown on CD exhibited a fruiting body production and a biological efficiency apparently higher than poplar chips-wheat straw (PC-WS) or wheat straw-wheat bran (WS-WB). On the other hand, *P. cornucopiae* showed not significantly higher production and biological efficiency compared to the WS (Figure 2a,b).

### 2.4. Structural Evaluation of Substrates after Fruiting Body Production

Gaussian curve-fitting procedure applied to the substrates ATR-FTIR spectra (CD, corn digestate-wheat straw-wheat bran (CD-WS-WB) and wheat straw-wheat bran (WS-WB)) after production of the fruiting body provided additional quantitative results. The area under the entire band was considered as 100%, and each component after fitting was expressed as a percent area. *Cyclocybe aegerita* was not included in the spectroscopic analyses, because its mycelium did not completely colonize the substrate and the fruiting body production was low.

The percentage area of each functional group can be considered representative of the structural modification as a consequence of the fruiting body production.

**Figure 3:**
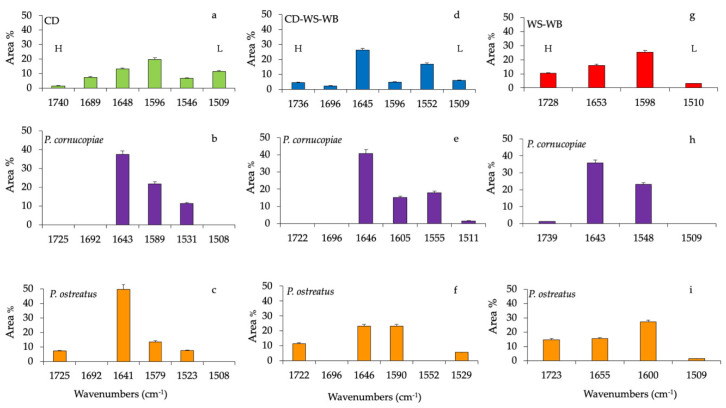
Histograms of peak areas (%) processed by using a curve fitting in the region from 1800 to 1200 cm^−1^. The best-fitting parameters were determined by the minimization of the reduced chi-square (χ^2^) and coefficients of determination (R^2^) that ranged from 0.990 to 0.980. The bar corresponds to the standard error. (**a**) Corn digestate (CD, green), (**d**) corn digestate-wheat straw-wheat bran (CD-WS-WB, blue) and (**g**) wheat straw-wheat bran (WS-WB, red) correspond to untreated substrates. Substrates after the fruiting body production of *P. cornucopiae* (purple) on (**b**) CD, on (**e**) CD-WS-WB, on (**h**) WS-WB and of *P. ostreatus* (orange) on (**c**) CD, on (**f**) CD-WS-WB and on (**i**) WS-WB. **H** = hemicellulose and **L** = lignin (as references for lignin: [29,30,31]).

Histograms of the percentage area of each considered band of CD, CD-WS-WB and WS-WB substrate spectra of untreated and after the fruiting body production of *P. ostreatus* and *P. cornucopiae* are shown in Figure 3. The ester in hemicellulose (1740 cm^−1^) in the untreated CD accounts for 1.4%, while it completely disappeared in the substrates with *P. cornucopiae* and, at the same time, represented 7.1% with *P. ostreatus.*

The band at 1680 cm^−1^ (C=O-conjugated ketone stretch) accounted for 7.3% and was only present on untreated CD (Figure 3a). The protein content in the CD was evaluated by amide I (1648 cm^−1^) and amide II (1546 cm^−1^), which accounted for 11.3% and 6.5%, respectively. In the composition of anaerobic digestate, the presence of bioactive substances such as proteins or amino acids was clearly identified [36]. Besides, the variation of the band at 1648 cm^−1^ may be also due to the deformation vibration of adsorbed water [25].

On the contrary, both amide groups increased considerably by 49.8% and 7.5% in the substrate, with *P. ostreatus* at 37.4% and 11.3% with *P. cornucopiae*, respectively. In the CD substrate, lignin bands (~1580 cm^−1^ and 1508 cm^−1^) contributed to 20% and 11.5%, respectively. After the fruiting body production of *P. ostreatus* and *P. cornucopiae,* the band at 1508 cm^−1^ disappeared completely, while the band at ~1580 cm^−1^ accounted for 13.5% with *P. ostreatus* and 21.8% with *P. cornucopiae* (Figure 3b,c). Variations in lignin peaks would indicate the opening of the lignin reticles through the removal of lignin subunits.

In the untreated CD-WS-WB substrate (Figure 3d), the CD presence could be recognized from the C=O conjugated ketone (1690 cm^−1^), amide I (1645 cm^−1^) and amide II (1550 cm^−1^) bands. All these functional groups were, respectively, 2.2%, 26.2% and 16.7%. As previously mentioned in the CD substrates, the band at 1690 cm^−1^ disappeared with *P. ostreatus* and *P. cornucopiae* (Figure 3e,f). In the substrate with *P. cornucopiae,* the contents of amide I and amide II were, respectively, 40.8% and 17.9%. The high value of amide I may also be due to the influence of the absorbed water [25]. On the other hand, in *P. ostreatus,* amide I decreased by 23%, while amide II was missing. The ester in hemicellulose (~1730 cm^−1^) in the untreated CD-WS-WB accounted for 4.5%; on the contrary, in *P. ostreatus*, it increased by 11.4%, and it was missing in *P. cornucopiae*. Regarding the lignin in the untreated substrate (~1596 cm^−1^ and 1509 cm^−1^), it accounted for 4.8% and 6.0%, respectively.

As a result of the production of fruiting bodies, lignin components with *P. ostreatus* changed only for the band at 1590 cm^−1^ by 23%, but no variation of the band at 1529 cm^−1^ (5.6%) was observed. In the substrates with *P. cornucopiae,* the band at 1605 cm^−1^ was around 15%, and the band at 1511 cm^−1^ was 1.5%. The delignification process in the substrate with *P. ostreatus* led to an increase in aromatic rings (1590 cm^−1^) and to the formation of new carbonyl groups in hemicellulose esters [29]. In the substrates with *P. cornucopiae,* lignin degradation was associated with the disappearance of the bands at 1509 cm^−1^ arising from the aromatic skeletal vibration of the benzene ring and, also, that of hemicellulose.

In the untreated WS-WB substrate (Figure 3g), the ester in hemicellulose was 10.3%, and the lignin accounted for 25% and 3%, respectively. After the fruiting body production of *P. cornucopiae* (Figure 3h) and *P. ostreatus* (Figure 3i), the hemicellulose accounted for 1.3%, and 14.7%, respectively. The lignin was 27% (1600 cm^−1^) and 1.6% (1508 cm^−1^) with *P. ostreatus* and 23% (1548 cm^−1^) with *P. cornucopiae.* As already observed in the CD-WS-WB substrate, the delignification and degradation process involved the same compounds.

## 3. Discussion

The anaerobic digestate from biogas plants has considerable operational and environmental drawbacks caused by the releasing greenhouse gases (NH_3_, CO_2_ and N_2_O) and by the presence of nondigested organic compounds [37,38,39]. The management of digestate with mushrooms that can decay the lignocellulosic component is creating a new scenario in biomass recycling in agriculture [12].

In this work, the mycelial growth in vitro and the biological efficiency of different mushroom species cultivated on CD-based substrates were explored. The structural modifications, as a result of the growth of different mushroom species, were monitored by using ATR-FTIR spectroscopy.

The results of the tests in the Petri dishes (Table S1) showed that *P. ostreatus* and *M. importuna* exhibited a higher growth rate on CD than on WS used as a control. Although these species are characterized by varying nutritional needs, the alkaline pH of the digestate has encouraged the growth of *M. importuna* [40]. Furthermore, for *P. ostreatus*, the higher nitrogen content in the CD than in the WS has contributed to a faster development, as already demonstrated in other studies [41,42] where a higher yield was obtained with straw that was enriched with nitrogen-rich plant supplements. As already shown by Santi et al. [43], the CD substrate would be suitable for the growth of lignivorous species such as *Pleurotus* spp. compared to digestates obtained from other biomasses.

The changes in substrate compositions in terms of elemental C and N (Table 1) after the mycelial growth in Petri dishes have shown an overall increase in the C/N ratio in the CD-WS and, especially, in the WS. Similar results were previously obtained in other works after 12 days of mycelial growth [44].

The variations observed in the WS may be due to the low C/N ratio of the durum wheat straw used in this trial. However, the biomasses from agrowastes are characterized by a great variation of the N amount and, thus, of the carbon-to-nitrogen (C/N) ratios. A considerable variation in the C/N ratio may also be assumed when the biomass is stored under aerobic conditions, as in our experiment, and consequently, an undesirable decomposition is expected. It was also reported that residues from crops with a C/N ratio below 30 should lead to a net N mineralization, while residues with a C/N ratio above 30 should promote immobilization [45].

In most cases, fungi are stimulated to incorporate N into proteins when the C source is lower than the N source. This could explain the significant reduction of N and the variation of amide bands in the FT-IR spectra of WS after mycelial growth.

The increase in total C may be explained as a combination of depolymerization of the lignocellulosic component and conversion of the labile C into the mycelial biomass. Generally, mushrooms in the early stages of lignin decomposition degrade it into a more labile component, and consequently, an apparent increase in the recalcitrant fraction may be observed [46]. All these variations are associated to structural alterations, as it was observed in our 12 days-study [46]. The structural variations of the substrates were dependent on the biomass type and mushroom species. After 12 days of mycelial growth, all tested species showed to be able to initiate depolymerization and, subsequently, decay the lignocellulosic component of CD. For example, in this early stage, *C. aegerita* and *C. comatus* degraded hemicellulose (1740 cm^−1^) and proteins (1647 cm^−1^ and 1547 cm^−1^), as demonstrated by the significant decrease in the relative intensity of the bands. Concerning lignin decay (1600 cm^−1^ and 1512 cm^−1^), *M. importuna* and both *Pleurotus* spp. were more active compared to *C. comatus* and *C. aegerita*. Recent studies carried out on *M. importuna* genome have shown that the laccase-like multicopper oxidase (LMCO) gene was present in the genome and may therefore support the observed effects [17].

In the case of the CD-WS, the substrate modifications were very similar to those observed for the CD with the involvement of *C. aegerita* and *C. comatus* for hemicellulose and protein degradation and both *Pleurotus* and *M. importuna* for lignin. As for the WS substrate, all species were able to modify it—in particular, *P. cornucopiae* and *P. ostreatus*, which attacked lignin more readily than hemicellulose.

Cultivation trials conducted on *C. aegerita*, *P. cornucopiae* and *P. ostreatus* highlighted the suitability of CD for the cultivation of edible mushrooms. Their biological efficiency on CD (19%, 80% and 103.3%, respectively) was in-line with those previously obtained on other substrates [42,47]. *Pleurotus ostreatus* and *P. cornucopiae* showed good biological efficiency, proving capable of exploiting digestate based substrates, as shown in previous studies conducted on *Pleurotus* spp. [48,49,50,51,52]. In particular, *P. ostreatus* grown on the CD exhibited a higher production of fruiting bodies (34% more) and biological efficiency (9% more) than the WS, the most used substrate for its cultivation [20,53,54]. The CD allowed to obtain a good fruiting body’s production of *Pleurotus* without the addition of the WB for its high N content (Table 1). In fact, the WB is commonly added to the WS as a nitrogen supplement for increasing the production [55].

Results on spent substrates after the production of the fruiting bodies showed a different decay of lignocellulosic constituents depending on the mushroom species. The most important change was observed for hemicellulose and lignin, which were completely decayed in the spent substrate of *P. cornucopiae*. Results showed that *P. cornucopiae* performed an efficient degradation of the lignocellulose component, particularly with only the digestate. Nevertheless, the constant presence of hemicellulose residues in all spent substrates of *P. ostreatus* was unexpected, although an increase in the cellulose content after mushroom growth on the WS was also reported [43,56]. Moreover, lignin decomposed in the CD and WS-WB-spent substrates. That is coherent with the activity of white-rot mushrooms. These mushrooms generate a mixture of ligninolytic enzymes that catalyze the oxidation of aromatic substrates by producing aromatic radicals and modifying the structure of biomasses that contain lignocellulose and lignin [51]. White-rot mushrooms carry out delignification through two main patterns: nonselective delignification or selective delignification [57]. In nonselective delignification, the degradation of lignin, cellulose and hemicellulose occurs simultaneously. Selective delignification, typical of different species of fungi, including *Pleurotus* spp., involves the degradation of lignin and hemicellulose before the attack of cellulose [57,58,59]. However, the chemical analyses in this study revealed a nonselective delignification for our strain of *P. cornucopiae*.

After lignin removal, spent substrates may be suitable for biogas production, as the materials are biologically predigested and are more available for bacterial degradation through anaerobic digestion [60,61,62]. Several works have underlined that pretreating different lignocellulosic biomasses with *Pleurotus* spp. before feeding them into the biogas plant leads to higher biogas production compared to the untreated biomasses with mushrooms [63,64,65,66]. Additionally, soil fertilization with the spent digestate obtained after mushroom growth produces benefits to the soil microbial communities from the availability of the released nutrients [67]. Consequently, the effects of fungi can be a powerful protective tool against soil fungal diseases [68].

The results obtained confirm the possibility to economically utilize the digestate for mushroom cultivation. Moreover, lignin removal by mushroom mycelium improves the degradation of hemicellulose and cellulose, and this is one of the strategies needed to improve its further utilization as fertilizer and opens up the possibility to obtain new biobased products.

## 4. Materials and Methods

### 4.1. Substrates

The corn digestate was obtained from a biogas plant that is located in Malalbergo (Bologna, Italy). The biogas plant can be classified as “single-phase, two-stage” and is designed to work in semi-dry conditions (total solid 13%) and is fed exclusively with energy crops (maize, sorghum and triticale silage). This plant can be considered to be of compact dimension compared to other technical solutions with the same productive capacity (4100 m^3^ of volume for 1 MW of installed electrical power), due to the ability to develop the process with high concentrations of substrate. The average biomass consumption is approximately 18,500 T/year for a biogas production of 4,050,000 m^3^ (8,500,000 kWh/year of gross renewable electric energy production). The mean hydraulic retention time (HRT) of the biomass is 70 days—after which, the digestate is separated into solid and liquid fractions through a mechanical screw separator. The solid digestate (SD) production is 2500 T/year and, after separation, is stored in trench silos similar to those commonly used for silomais. The liquid digestate (10,000 T/year) is stocked in a specifically covered tank. The SD used in this work was produced in February and collected as soon as it fell out of the mechanical separator.

Durum wheat straw and *Populus* spp. chips were kindly provided by the Cadriano farm of the University of Bologna. In particular durum wheat straw was used in the farm to produce compost, and it was stored under aerobic conditions in the field. All raw materials were dehydrated at room temperature and kept in the stove at 60 °C for 24 h. They were subsequently crushed with scissors and a manual grinder into fragments smaller than 0.5 cm and autoclaved at 121 ± 1 °C for 60 min to prevent any contamination during their storage. The dried raw materials were stored in a desiccator at 22 °C.

Organic soft wheat bran and Alabastrine gypsum (CaSO_4_·2 H_2_O, lab grade) were used for the mushroom cultivation trials.

### 4.2. Mushrooms Cultures and Mycelial Growth Rate Evaluation

Experimental trials were carried out by using 9 strains of Basidiomycetes (*C. aegerita*, *C. comatus*, *G. lucidum*, *G. resinaceum*, *L. sulphureus*, *P. cornucopiae*, *P. ostreatus* and *S. commune*) and Ascomycetes (*M. importuna*) isolated from fruiting bodies collected in the wild; the *L. edodes* (Basidiomycete) strain was brought by the Fungal Institute of Jinxiang (Shan-dong province, China) (Table S1).

The mycelial pure cultures were stored in the Mycological and Applied Botany Laboratory (CMI-UNIBO strain collection) of the Department of Agricultural and Food Sciences (DISTAL), University of Bologna (Italy).

All the isolates were kept on potato dextrose agar (PDA, Difco, Detroit, MI, USA) half-strength, at 22 ± 1 °C in darkness and subcultured every two months.

For the mycelial growth rate evaluation, plugs of 10 mm in diameter were taken by 15-day-old colonies of each species and inoculated in the center of a 9-cm sterilized Petri dish previously filled with 15 g of three different substrates at 80% humidity: CD, CD-WS (1:1, *w/w*) and WS as the control. Five replicates were made for each combination of species and substrate. All plates were incubated at 22 ± 1 °C in darkness. The mushroom growth was assessed by measuring the diameter of the colony along two preset diametrical lines every day. Radial daily growth rate (rGR) was calculated during the first 3 days of the exponential phase as:

$$\mathrm{rGR}=\frac{{(D}_{1}{\;–\;D}_{0})\;/\;2}{{(t}_{1}{\;–\;t}_{0})}\;\times \;24$$

where D_0_ and D_1_ are the colony diameter during the exponential growth phase at time t_0_ and t_1_, respectively [69] (modified).

### 4.3. Inoculum Preparation and Cultivation Trials

Cultivation tests were carried out for *C. aegerita*, *P. cornucopiae* and *P. ostreatus*. The grain spawn was prepared by inoculating 15-day-old mycelial plugs in glass tubes containing sorghum kernels and distilled water in a 1:2 (*v/v*) ratio previously sterilized at 121 ± 1 °C for 20 min. For each species-substrate combination, 5 replicates were prepared. The tubes were incubated in the dark at 22 ± 1 °C for 30 days. The spawn was ready when the mycelium had colonized all the kernels.

Three different solid substrates were tested: a digestate based substrate (97% corn digestate), a substrate with 48% of digestate and as a control and a substrate based on poplar chips (PC) or WS according to the mushroom substrates preferences [20,55] (Table 2). Homogeneous substrate mixtures were prepared by mixing component materials based on their dry weight (*w/w*).

The substrates were inserted into autoclavable polypropylene transparent bags (20 × 30 cm). Dried mixed substrates (1.5 L per bag: 120 g for CD, 110 g for CD-WS-WB and 100 g for WS-WB and 160 for CD-PC-WS and 200 g for PC-WS) were moistened with 500 mL (per bag) of distilled water for 24 h, and the excess of water was removed by squeezing the bags.

Bags were closed with a hydrophobic cotton cap, autoclaved at 121 ± 1 °C for 60 min and inoculated with grain spawn by opening the bag and inserting the inoculum inside of it (45 g per bag), mixing the inoculum with the substrate and incubating it in the darkness at 22 ± 1 °C until complete colonization of the substrate. For each species-substrate combination, five bags were prepared. Mycelial bag colonization was evaluated.

After 20 or 35 days of mycelial growth of the two *Pleurotus* species and *C. aegerita*, respectively, the bags were moved to a climatic chamber with a temperature of 19 ± 1 °C during the day and 14 ± 1 °C during the night, relative humidity between 80% and 85% and 12-h light/dark photoperiod with a light intensity of 700 ± 100 lux. The mature fruiting bodies were collected for 3 months; the fresh weight was recorded for the evaluation of biological efficiency [70]; then, they were dried in a stove at 65 °C for 24 h and weighed. Biological efficiency was calculated as:

$$\mathrm{Biological}\;\mathrm{efficiency}\;\left(\%\right)=\frac{\mathrm{fresh}\;\mathrm{weight}\;\mathrm{of}\;\mathrm{mushrooms}}{\mathrm{dry}\;\mathrm{weight}\;\mathrm{of}\;\mathrm{substrate}}\times 100$$

### 4.4. Chemical Analyses

In order to evaluate changes in the chemical composition of the different substrates before and after the 12 days of mycelial growth, the elemental analysis of C and N was carried out. Five replicates of each substrates before and after inoculation were collected from the Petri dishes. All samples were mixed, washed five times with distilled water, dried in a stove at 35 °C for 48 h and crushed in a ball mill (RETSCH MM 400, Haan, Germany) to produce a homogeneous mixture of each material. The elemental analysis was carried out using Flash EA 2000 Elemental Analyzer (Thermo Scientific, Bremen, Germany) on 2 mg and performed on triplicate samples.

### 4.5. Attenuated Total Reflectance-Fourier Transform Infrared (ATR-FTIR) Spectroscopy Analysis

The FTIR spectra were recorded by using an ALPHA FTIR spectrometer (Bruker Optics, Ettlingen, Germany) equipped with a diamond crystal ATR (attenuated total reflectance) device. The substrates were analyzed after 12 days of in vitro mycelial growth and after the fruiting bodies production (4 months after inoculation) were analyzed. Each sample was deposited on the surface of the crystal, and the spectra were acquired against a pre-established background by averaging 64 scans from 4000 to 400 cm^−1^ at a 4-cm^−1^ resolution. Spectra were collected in triplicate for each sample and then averaged.

In order to determine the main structural changes in the spectra that had undergone different treatments, a curve fitting using the Grams/386 spectroscopic software (version 6.00, Galactic Industries Corporation, Salem, NH, USA) was performed. The second derivative of the IR spectra in the 1800–1200 cm^−1^ region was smoothed using the Savitzky–Golay function. The IR spectra were fitted with Gaussian bands, and the best-fitting parameters were determined by minimization of the reduced chi-square (χ^2^). Agreement between the experimental and calculated profiles was obtained, with coefficients of determination, R^2^, ranging from 0.990 to 0.980 and the standard error, SE, from 0.001 to 0.005.

### 4.6. Statistical Analyses

Statistical analyses were assessed by using the XLSTAT software version 7.5.2 (Addinsoft, New York, NY, USA). Student *t*-test was performed to compare the C and N contents of the substrates after the mushrooms’ mycelial growth with the initial substrates. The analysis of variance (ANOVA) was used to determinate a significant difference in the mycelial growth rate, fruiting body production and biological efficiency among different substrates. Tukey post hoc test (*p* ≤ 0.05) was used to compare the means.

## Figures and Tables

**Table 1:** Total content of C and N on different substrates before inoculation with the fungi studied (control) and after a 12-d period of mycelium growth following inoculation.

Species	Substrates					
	CD		CD-WS		WS	
	C (%)	N (%)	C (%)	N (%)	C (%)	N (%)
Control	39.07 ± 0.07	1.59 ± 0.01	39.57 ± 1.04	1.18 ± 0.005	36.26 ± 0.005	1.16 ± 0.04
*Cyclocybe aegerita*	38.67 ± 0.02 **	1.54 ± 0.04	39.18 ± 0.004	1.13 ± 0.01 *	37.93 ± 0.99	0.62 ± 0.008 **
*Coprinus comatus*	39.60 ± 0.47	1.51 ± 0.07	39.43 ± 0.18	1.15 ± 0.01	39.55 ± 0.27 **	0.67 ± 0.01 **
*Morchella importuna*	38.94 ± 0.04	1.55 ± 0.04	39.81 ± 0.29	1.08 ± 0.02 **	39.86 ± 0.79 *	0.68 ± 0.02 **
*Pleurotus cornucopiae*	39.07 ± 0.05	1.37 ± 0.10	38.80 ± 0.32	1.20 ±0.04	38.52 ± 0.05 **	0.61 ± 0.01 **
*Pleurotus ostreatus*	39.86 ± 0.59	1.59 ± 0.05	39.39 ± 0.96	0.81 ± 0.007 **	39.61 ± 0.68 **	0.57 ± 0.009 **

Corn digestate—CD, corn digestate 50%-wheat straw 50%—CD-WS and wheat straw—WS. The data are the mean of 3 replicates ± standard error. Asterisks indicate significant differences from the control (* *p* ≤ 0.05 and ** *p* ≤ 0.01) using a Student’s *t*-test.

**Table 2:** Composition of the substrates used for cultivation trials.

Species	Substrates and Mixing Ratio ^1^	Code
*P. cornucopiae* *P. ostreatus*	97% CD:3% gypsum	CD
	48% CD:46% WS:3% WB:3% gypsum	CD-WS-WB
	92% WS:5% WB:3% gypsum (control)	WS-WB
*C. aegerita*	97% CD:3% gypsum	CD
	48% CD:44% PC:5% WS:3% gypsum	CD-PC-WS
	88% PC:9% WS:3% gypsum (control)	PC-WS

^1^ dry weight (*w*/*w*). Abbreviations: CD—corn digestate; WS—wheat straw; WB—wheat bran and PC—poplar wood chips.

## Supplementary Materials

Figure S1: Growth of (**1**) *C. aegerita*, (**2**) *C. comatus*, (**3**) *G. lucidum*, (**4**) *G. resinaceum*, (**5**) *L. sulphureus*, (**6**) *L. edodes*, (**7**) *M. importuna*, (**8**) *P. cornucopiae*, (**9**) *P. ostreatus* and (**10**) *S. commune* in Petri dishes on (**a**) wheat straw (WS, red line), (**b**) corn digestate 50%-wheat straw 50% (CD-WS, blue line) and (**c**) corn digestate (CD, green line). Figure S2: Fruiting body production of (**a**,**b**) *P. ostreatus* and (**c**,**d**) *P. cornucopiae*. Table S1: Radial growth rate (mm/day) of the tested mushroom species on different substrates. Table S2: C/N ratio on different substrates before and after inoculation with the mushrooms.
