# Supplementary material for: Degradative Ability of Mushrooms Cultivated on Corn Silage Digestate
Source: Molecules. 2020 Jul 1;25(13):3020. doi: 10.3390/molecules25133020 (PMC7412174; doi:10.3390/molecules25133020)
Supplement: Supplementary file 1 [file molecules-25-03020-s001.zip › Supplemental materials/Table S2.docx]

**Table S2.** C/N ratio on different substrates before and after inoculation with mushrooms.

| **Species** | **Substrates** | | |
| --- | --- | --- | --- |
|  | **CD** | **CD-WS** | **WS** |
|  | **C/N** | **C/N** | **C/N** |
| Control | 24.57 | 33.53 | 31.26 |
| *C. aegerita* | 25.11 | 34.54 | 61.16 |
| *C. comatus* | 26.20 | 34.29 | 59.03 |
| *M. importuna* | 25.12 | 37.21 | 58.62 |
| *P. cornucopiae* | 28.51 | 33.16 | 63.15 |
| *P. ostreatus* | 25.23 | 48.63 | 69.49 |

Abbreviations: Corn Digestate—CD, Corn Digestate 50%-Wheat Straw 50%—CD-WS and Wheat Straw—WS.
