# Supplementary material for: Degradative Ability of Mushrooms Cultivated on Corn Silage Digestate
Source: Molecules. 2020 Jul 1;25(13):3020. doi: 10.3390/molecules25133020 (PMC7412174; doi:10.3390/molecules25133020)
Supplement: Supplementary file 1 [file molecules-25-03020-s001.zip › Supplemental materials/Table S1.docx]

**Table S1.** Radial growth rate (mm/day) of the tested mushroom species on different substrates.

| **Strain n.*** | **Species** | **CD** | **CD-WS** | **WS** |
| --- | --- | --- | --- | --- |
| CAe4 | *Cyclocybe aegerita* | 5.34 ± 1.22 A | 4.77 ± 1.04 A | 5.33 ± 0.31 A |
| CCo10 | *Coprinus comatus* | 2.46 ± 0.64 B | 9.26 ± 0.76 A | 8.24 ± 1.15 A |
| GLu16 | *Ganoderma lucidum* | 0.32 ± 0.10 B | 0.38 ± 0.21 B | 4.08 ± 0.09 A |
| GRe5 | *Ganoderma resinaceum* | 1.37 ± 0.45 C | 5.4 ± 0.55 B | 8.18 ± 0.92 A |
| LSu10 | *Laetiporus sulphureus* | 0.00 B | 0.11 ± 0.16 B | 6.74 ± 1.24 A |
| LEd5 | *Lentinula edodes* | 0.00 B | 0.59 ± 0.57 B | 2.82 ± 0.41 A |
| MIm6 | *Morchella importuna* | 21.50 ± 1.00 AB | 23.50 ± 3.09 A | 20.20 ± 1.01 B |
| PCo3 | *Pleurotus cornucopiae* | 8.18 ± 0.68 A | 8.37 ± 0.71 A | 7.88 ± 0.86 A |
| POs15 | *Pleurotus ostreatus* | 4.83 ± 0.75 A | 4.10 ± 1.60 A | 2.25 ± 0.33 B |
| SCo3 | *Schizophyllum commune* | 6.59 ± 1.72 C | 8.52 ± 0.33 B | 10.60 ± 0.20 A |

***** Strain collection CMI-UNIBO. Corn Digestate—CD, Corn digestate 50%-Wheat straw 50%—CD-WS and Wheat Straw—WS. The data are the mean of 10 measurements ± standard deviation. Different letters on the same row indicate significant difference for *p* ≤ 0.05 by post-hoc Tukey test.
