# Supplementary figures and images for: Degradative Ability of Mushrooms Cultivated on Corn Silage Digestate
Source: Molecules. 2020 Jul 1;25(13):3020. doi: 10.3390/molecules25133020 (PMC7412174; doi:10.3390/molecules25133020)

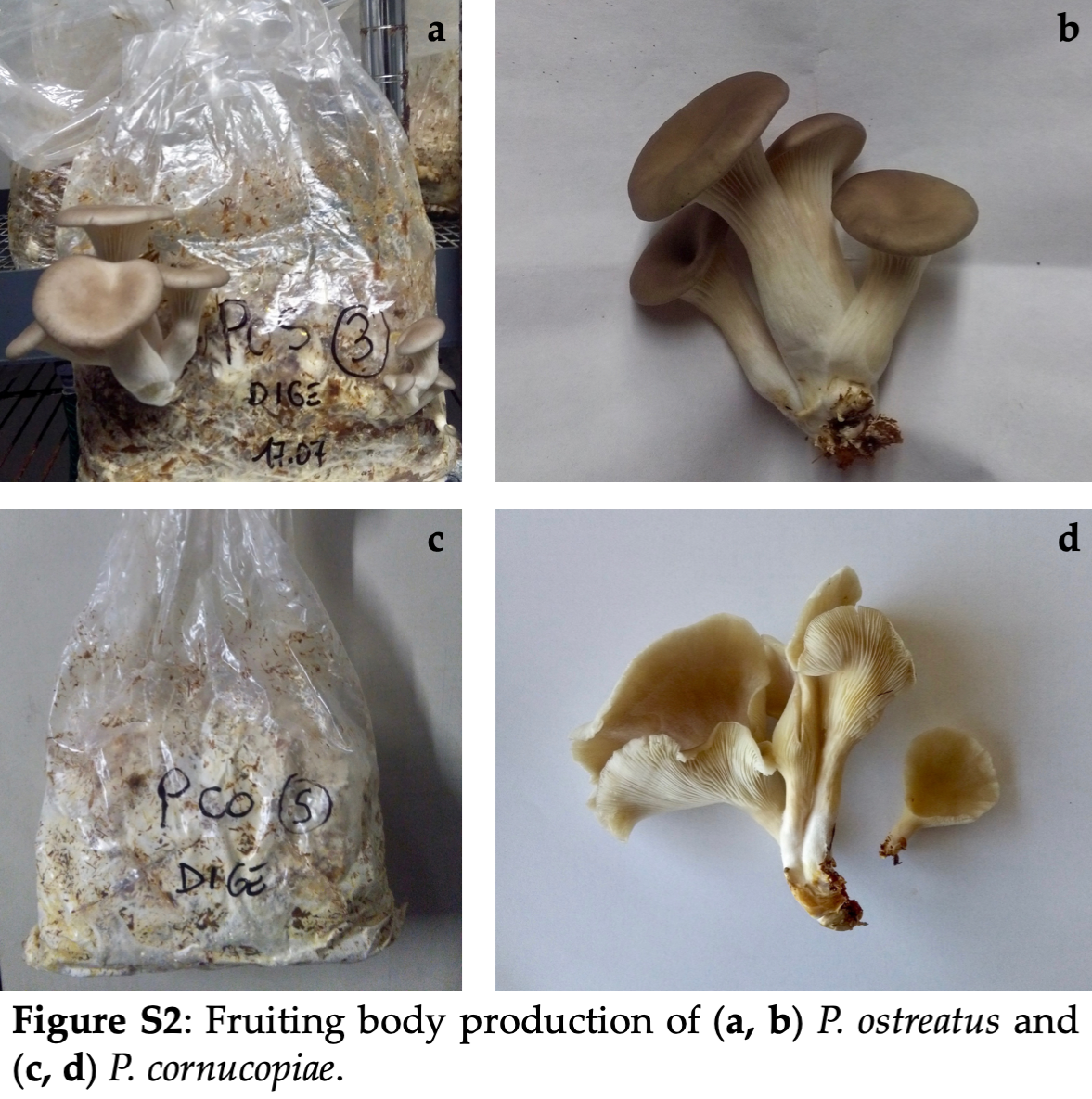

Supplement: Supplementary file 1 [file molecules-25-03020-s001.zip › Supplemental materials/Figure S2.tiff]

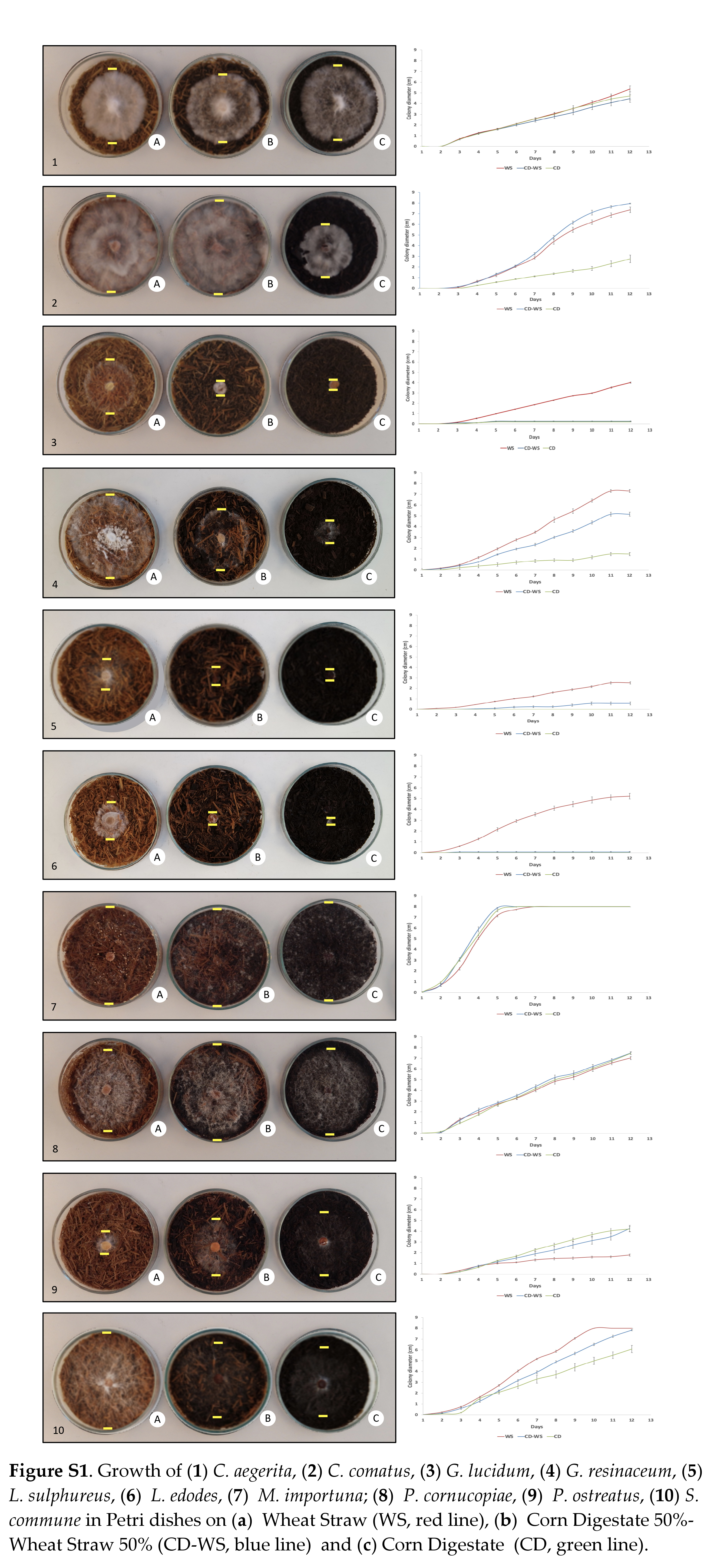

Supplement: Supplementary file 1 [file molecules-25-03020-s001.zip › Supplemental materials/Figure S1.jpg]
